# The Influence of Internal and External Stakeholder Mechanisms on Entrepreneurial Success: The Moderating Role of Digital Technology Adoption

**DOI:** 10.3389/fpsyg.2021.821725

**Published:** 2022-02-03

**Authors:** Cui Yong, Saba Fazal Firdousi, Ayesha Afzal, Viktorija Florjančič, Minahil Awais

**Affiliations:** ^1^Overseas Education College, Jiangsu University, Zhenjiang, China; ^2^Lahore School of Economics, Lahore, Pakistan; ^3^University of Primorska, Koper, Slovenia

**Keywords:** family support, business partner support, community support, external stakeholder relationships, digital technology adoption, entrepreneurial success, integration amongst education-industry-government, China

## Abstract

The purpose of this research is to investigate the associations of internal and external support mechanisms with entrepreneurial success, in the context of China's entrepreneurial sector from network theory perspective. The role of digital technology, as a moderator, has also been analyzed. Data has been obtained from 500 entrepreneurs in Jiangsu, a province in China. All hypotheses were tested using structural equation modeling. It has been found that family support, business partner support, community support and external stakeholder relationships have positive effects on entrepreneurial success. It has also been discovered that digital technology adoption strengthens the positive relationship between business partner support and entrepreneurial success. Theoretical and practical implications have been highlighted and future research suggestions have been provided.

## Introduction

Digital technology is becoming increasingly important in the business world (Gupta and Jain, 2012; Bharadwaj et al., 2013; Matt et al., 2015; Sorescu, 2017). It can promote entrepreneurship (Linton and Solomon, 2017) and help achieve entrepreneurial success (Venkatesh et al., 2017). In this context, China is overtaking the world as the new Silicon Valley (Klingler-Vidra, 2019). In the last decade the Asian country has experienced rapid and dynamic growth in entrepreneurship (Hemmert et al., 2021). China is making efforts to support innovation by businesses and planning to support 1 million innovative small and medium enterprises by 2025, according to a government guideline (Huaxia, 2021a). It is now amongst the few nations in the world that possess several internationally competitive start-up ecosystems (Barton et al., 2017). Currently, China ranks second globally with 301 unicorn companies or start-ups valued at more than US$ 1 billion, in 2021 (Huaxia, 2021b). Over the last year, 74 new Chinese unicorn companies have been added to the list (Global Unicorn Index 2021). Chinese tech giant Bytedance is the most valuable unicorn in the world, with its value equal to around US$ 350 billion (Huaxia, 2021b). Importantly, the country has active use of technology in business operations (Hou et al., 2020).

Entrepreneurship is a driver of economic growth which means it can help developing economies (Christensen et al., 2009; Kimmitt et al., 2020). So, it is important to understand the determinants of entrepreneurship (Acs et al., 2008; Carree and Thurik, 2010; Gries and Naudé, 2010). However, there is a lack of knowledge about what boosts entrepreneurship in developing countries (Bruton et al., 2008; Chatterjee et al., 2018). Our knowledge about young entrepreneurs in developing economies is also limited (Manolova et al., 2019) even though most of the people who start a business are 25–34 years old (Levesque and Minniti, 2006; Lévesque and Minniti, 2011) and most individuals under 30 live in developing economies (United Nations Educational, Scientific and Cultural Organization (UNESCO), 2013).

These people are significantly more entrepreneurship-minded, compared with older generations (Salkowitz, 2010). They can also use their networks to generate new value and wealth. In addition, young entrepreneurs are tech-savvy which is favorable for entrepreneurship (Manolova et al., 2019). Research has shown that, amongst developing economies, China has a larger segment of entrepreneurs who have contributed to significant economic progress (Ahlstrom and Ding, 2014; Su et al., 2015). Further, expansion of the entrepreneurship sector in China's economy is an important driver of sustainable economic growth (Gok et al., 2021). These facts make China the right context for fruitful entrepreneurship research.

In this topic, research states that a business is embedded in a network of relationships from which it can obtain essential resources (Andersson and Forsgren, 1996; Andersson et al., 2002). Support from these network partners can promote entrepreneurship (Van de Vrande et al., 2009; Huggins and Thompson, 2017; Elia et al., 2020). After an enterprise is created, its potential for survival and growth may depend on the ability of the entrepreneur to obtain resources (Zhao and Aram, 1995). Specifically, it is beneficial to use internal and external sources to acquire a competitive advantage through technology (Zahra and Nielsen, 2002). Aligned with this, research has revealed that networking can boost business growth and success (Jarillo, 1988, 1989; Chell and Baines, 2000; Huggins, 2000). In this area, there is a lack of understanding of whether the associations hold in developing economies (Soluk et al., 2021) so more research is required. More research is also needed about the impact of family support on entrepreneurial success (Staniewski and Awruk, 2021).

Since the new businesses in which venture capital is invested have little to show in terms of past performance, the capitalists rely on social ties to assess entrepreneurs and their ideas (Sorenson and Stuart, 2001). Further, a popular method for increasing business innovation is participation in networks that are designed to realize potential synergies (Hagedoorn and Schakenraad, 1994; Human and Provan, 1997; Wincent, 2008; Thorgren et al., 2009; Wincent et al., 2009, 2010). Maintaining these ties with multiple stakeholders provides an organization with relevant information for more proactive entrepreneurship behavior (Vandekerckhove and Dentchev, 2005).

Another relevant factor for business is technology because most enterprises today are, and will continue to be, affected by digitalisation (Nambisan, 2017; Kammerlander et al., 2018; Soluk and Kammerlander, 2021). Digital technologies promote new businesses (von Briel et al., 2018a) and the number of entrepreneurs engaging with these technologies is increasing quickly (Morse et al., 2007). Entrepreneurs adopt technologies so that they can utilize market opportunities (Audretsch and Link, 2012). This process is widely expected to boost productivity (Stoneman and Kwon, 1996; Baumol and Strom, 2007; Boothby et al., 2010; Krammer, 2015). As digital enterprises are becoming more common, it is becoming more important to understand the opportunities and threats in digital entrepreneurship (Hansen, 2019). This is why there are calls for studies assessing how technologies impact innovation (Nambisan et al., 2017; Majchrzak et al., 2018).

China is a relevant context here because it is one of the world's largest developing economies and in the middle of digital reformation (Hansen, 2019). It had a deep-rooted denunciation of private enterprise and the Internet, which started changing in the 1990s and now the country has a strong private sector that provides multiple opportunities for entrepreneurship (Hansen, 2019). Further, digital technology has become necessary for business survival (Mazzarol, 2015). However, the country still has a low level of entrepreneurship so research is needed in this area (Krasniqi, 2014).

China's government has developed multiple policies to support entrepreneurship (Lavelle, 2021). For example, the government made it compulsory for higher education institutions to provide entrepreneurship education in 2015 (Qiang et al., 2016; Yu, 2018). Under this policy, students are equipped with entrepreneurial abilities so that they become an innovative workforce (Qiang et al., 2016). Another example is the Belt and Road Initiative which is a large-scale, long-term project started by the Chinese government, and with participation by other countries, to support economic development (Lee and Shen, 2020). This project has generated multiple innovation opportunities, including entrepreneurial development (Lee and Shen, 2020).

The key research objectives of this study are to explore influence of internal and external support mechanisms on entrepreneurial success. The research model is based on network theory. The support mechanisms are family support, business partner support, community support and external stakeholder relationships. Moreover, the existing literature have highlighted the role of digital technology in many areas. However, it is under explored in the context of internal and external support mechanisms. Therefore, we have examined digital technology as a moderating factor in the relationships of support mechanisms and entrepreneurial success.

Taking these factors into account, we have conducted cross-sectional research in the context of China. A sample of entrepreneurs was selected, using the convenience sampling method. A questionnaire was developed for them, using validated measuring tools for the constructs in our conceptual model. The data obtained were analyzed using structural equation modeling (SEM) in SmartPLS v3. This study has generated valuable findings that have both theoretical and practical implications.

This research has covered a gap in existing literature, by including both internal and external support mechanisms for entrepreneurial success. Specifically, the role of external stakeholder support needed to be analyzed (Soluk et al., 2021) so the study has made a theoretical contribution. Soluk et al. (2021) have analyzed the impact of multiple variables on entrepreneurship but they have used hierarchical regression analysis. Chen et al. (2015) have investigated the impact of different networks on entrepreneurial success but their model does not include the important role of technology adoption. We have applied the statistical technique partial least squares SEM (PLS-SEM) in the software SmartPLS v3, to analyse a detailed model. Recently, the number of published articles using this method has increased significantly, compared with covariance-based SEM (CB-SEM) (Hair et al., 2017b). We have selected the method because it is superior, in terms of statistical power, compared with CB-SEM (Reinartz et al., 2009; Hair et al., 2017b). This means that it is more likely to highlight relationships as significant when they are indeed present in the population (Sarstedt and Mooi, 2014). As far as we know, past studies have not applied these tools to analyse such a detailed model. So this study has made an empirical contribution also.

This research has made multiple conceptual contributions. First, it has added to the inadequate knowledge on how family support influences entrepreneurship success (Staniewski and Awruk, 2021). Second, the results are different from those obtained by Soluk et al. (2021) because a negative relationship between business partner support and entrepreneurship has not been found. This shows that business partner support (integration of education, industry and government) does not necessarily cause problems in the context of entrepreneurship. In fact, it acts as a concrete block for development of nascent entrepreneurs. Third, a detailed framework has been developed. As far as we know, such a model has not been explored in past studies. Fourth, the research has responded to calls for analyzing the effects of technology on relationships between support systems and entrepreneurial success (Nambisan et al., 2017; Majchrzak et al., 2018). Fifth, the overall model has provided support for network theory (Watson, 2007). This study has also made multiple contextual contributions. First, it has investigated entrepreneurship in a developing context (Bruton et al., 2008; Chatterjee et al., 2018). Second, it has added to the insufficient knowledge about digital technology's role in developing countries (Soluk et al., 2021).

The first practical implication is that this study has highlighted sources of support available for boosting entrepreneurial success. These include family members, community members, business partners and external stakeholders. Second, it has underlined the importance of digital technology in promoting entrepreneurial success. Third, the findings are useful for policy makers for a better understanding of entrepreneurship in developing economies. Using these results, appropriate strategies can be designed. In this context, digital transformation of a society can helps take developing economies to the next level (Soluk et al., 2021).

The next section of this article will present a review of relevant studies and hypotheses extracted from them. Then the methodology will be discussed. This includes the sample, measurement of variables and statistical tools. Then the results will be discussed and implications will be provided. Recommendations for future research will also be given. The paper will end with a conclusion.

## Literature Review, Theoretical Basis and Hypothesis Development

This study is based on network theory perspective. It states that the capability of owners to use networking for efficiently acquiring resources can increase the probability of entrepreneurship success (Zhao and Aram, 1995). Supporting this, research has found that entrepreneurs can obtain valuable resources through networking (Zhao and Aram, 1995) and that networking has positive relationships with business survival and growth (Watson, 2007). Multiple studies have found positive associations between organizational networking and performance (Watson, 2007). For example, Duchesneau and Gartner (1990) have discovered that successful organizations are more likely to have obtained professional consulting. Similarly, Potts (1977) has found that successful businesses rely more on accountants' advice. The financial performance of businesses has been found to be positively related to external management advisory services (Kent, 1994).

Donckels and Lambrecht (1995) have discovered that network growth leads to enterprise growth. Importantly, researchers have observed that networking is likely to be even more useful for new enterprises (Bruderl and Schussler, 1990; Stuart and Sorenson, 2003) because the young entrepreneurs who manage them lack resources and experience (Evans and Jovanovic, 1989; Nielsen and Lassen, 2012; Shirokova et al., 2017).

In China's culture, interpersonal relationships are considered very important (Tse et al., 1988). So networking is highly compatible with Chinese business customs and businesses revolve around reliable relationships (Zhao and Aram, 1995). The art of guanxi is essential for obtaining scarce resources (Brunner et al., 1989) in the absence of institutions, such as law (Zhao and Aram, 1995). Therefore, it is of significance to explore the influence of internal and external support mechanisms on entrepreneurial success with the moderating role of digital technology adoption from network theory perspective.

### Family Support and Entrepreneurial Success

An entrepreneur's family is an important stakeholder of the enterprise (Aldrich and Cliff, 2003; Bruque and Moyano, 2007; Duran et al., 2016; Hatak et al., 2016). Family inputs can be very useful because entrepreneurs may interact often with family members and this interaction is based on high levels of trust and understanding (Soluk et al., 2021). A family is made up of a diverse group of individuals, especially in developing countries where extended family members are also included in the definition (Dasgupta et al., 1999; Niranjan et al., 2005). Networking with family members of different ages, personalities and professions may provide entrepreneurs with valuable inputs for their ventures such as new ideas for improved products (Soluk et al., 2021).

Entrepreneurs can ask their families to provide time, effort, tangible assets or funding (Soluk et al., 2021). In developing economies, institutional gaps such as lack of reliable information systems often obstruct entrepreneurial start-ups and their success (Van Stel et al., 2007; Stenholm et al., 2013). Since there is a lack of resources in developing countries (Desai and Joshi, 2014) such as China, this support is needed to cross barriers and achieve success (Soluk et al., 2021).

In these developing economies, entrepreneurship ventures might be less complicated so family members are more likely to understand them well (Manrai and Manrai, 2001) and be able to contribute. Advice or encouragement, from families, can give individuals the boost they need to pursue entrepreneurship (Lalhunthara, 2019). Family members can act as mentors, or even role models, if they have an entrepreneurship background (Minniti and Bygrave, 1999; Aldrich and Cliff, 2003). Research on entrepreneurship often discusses the high probability of failure (Shane and Venkataraman, 2000) so in this context, an entrepreneur's family can provide emotional support.

It is also necessary to take into account the harmful effects of family support on entrepreneurial success. Initially, the negative association sounds strange but it makes sense if one considers that some societies can allow family members to interfere in the work, produce conflict and worsen problems (Welsh et al., 2014). Manolova et al. (2019) have found a negative relationship between family financial support and start-up actions. This is because the capital can be viewed as easy money or may carry certain conditions (Manolova et al., 2019). This unfavorable impact of networking on performance can be explained by the fact that family support is based on certain expectations and if these are not met, conflict can arise and affect entrepreneurship outcomes negatively (Xu et al., 2020). Based on the research discussed above, the following relationship has been hypothesized.

*H*_1_*: There is a positive relationship between family support and entrepreneurial success*.

### Business Partner Support and Entrepreneurial Success

Businesses benefit from building and maintaining long-term relationships (Powell et al., 2005; Kumar and Pansari, 2016). For example, partnerships allow sharing of research and development expenses (Turnbull and Leung, 1986; Danneels, 2002; Haeussler et al., 2012). Hellström (2004) has argued that innovation involves social actions where innovation lies in those exchanges. Supporting this, research has found a positive relationship between entrepreneurship and partner fit in business networks (Thorgren et al., 2012).

In this context an enterprise's ties with its customers and suppliers involve trust, information-sharing and problem-solving (Tsai and Wen, 2009) so are highly valuable. Building social ties with customers is required for mutual benefits, in terms of innovation (Dickson and Hadjimanolis, 1998; Pittaway et al., 2004; Morrissey and Pittaway, 2006). Research has found that it is beneficial for a new venture to include its suppliers in operations (Song et al., 2011). The rationale behind this is that networking with other businesses increases the probability of success as an organization can learn from partners and take advantage of their assets (Dowling and Helm, 2006). For high-technology ventures, cooperation is necessary for launching new products successfully (Dowling and Helm, 2006).

In this context of support, entrepreneurial education has been found to be effective for stimulating entrepreneurial intentions in China (Lavelle, 2021). In harmony with this, Cui et al. (2019) have found that entrepreneurship education has a positive effect on the entrepreneurial mindset. However, some past studies have also found that entrepreneurship education negatively affects entrepreneurial actions (Dou et al., 2019). So, findings on the entrepreneurial education-behavior relationship are mixed.

In developing countries, the scenario may be different. Since there is presence of institutional gaps such as unreliable legal systems (Welter and Smallbone, 2011), organizations may not trust long-term relationships with partners. This is why business partners show lower motivation to share entrepreneurship ideas (Soluk et al., 2021). In this context, Tsai and Wen (2009) have found that entrepreneurship in China has an inverted-U relationship with customer or supplier networks. Initially a venture gains benefits from networking but after a certain point, entrepreneurship starts being negatively affected. Based on the findings highlighted above, the following relationship has been hypothesized.

*H*_2_*: There is a positive relationship between business partner support and entrepreneurial success*.

### Community Support and Entrepreneurial Success

Belonging to a caring community helps generate more value through creativity (Ghezzi et al., 2018). A community can serve as the base for entrepreneurship operations and success (Hindle, 2010). Community networks have been found to increase entrepreneurship (Soluk et al., 2021), foster entrepreneurial growth (Sankaran and Demangeot, 2017) and help accomplish entrepreneurship success (McGehee et al., 2010; Bosworth and Farrell, 2011; Kwon et al., 2013). Further, trust in a community lowers the cost of building contracts and monitoring adherence (Kwon et al., 2013). This trust has a positive impact on entrepreneurial actions. In addition, community trust leads to information being shared about entrepreneurs who would otherwise have had little prominence (Kwon et al., 2013).

Communities provide support to members' enterprises and communication is easier with customers because there is familiarity amongst the members (de Guzman et al., 2020). Chandna and Salimath (2020) have found that when there is a sense of community, ventures spread more positive word-of-mouth for other organizations' products and provide more community support. Communities can also play the role of financial institutions, to decrease financial pressure (Lyons et al., 2012). Regions with more community networking benefit more when resources are provided for entrepreneurship (Samila and Sorenson, 2017).

In a developing economy, community initiatives can compensate for institutional gaps (Scott, 1995; Torri, 2010; Desai and Joshi, 2014; McAdam et al., 2019). In China entrepreneurs enrich and use their guanxi networks, which include community links, to acquire resources for their new ventures (Chen et al., 2015). This networking has a positive impact on entrepreneurs' success (Chen et al., 2015). Based on the research discussed above, the following relationship has been hypothesized.

*H*_3_*: There is a positive relationship between community support and entrepreneurial success*.

### External Stakeholder Relationships and Entrepreneurial Success

External stakeholders are defined as stakeholders outside an organization (Mazur and Pisarski, 2015). For entrepreneurial start-ups and their growth, these stakeholders must be viewed as sources of opportunity (Kuratko et al., 2007). Aligned with this, research has shown that information from third parties can help entrepreneurs in discovering opportunities (Kuratko et al., 2007) to improve business performance (García-Sánchez et al., 2018). In China, Qinghuai is a networking construct that is considered vital for success of digital entrepreneurship (Xiao et al., 2020). It is related to an organization's mission and its relationships with external stakeholders (Xiao et al., 2020).

Associations with stakeholders can positively impact entrepreneurship (Vershinina et al., 2020). Further, stakeholders play an important role in maintaining a competitive advantage (Shams, 2016a,b, 2017). Working in collaboration with stakeholders positively affects entrepreneurs' success (García-Sánchez et al., 2018). Research has provided multiple examples to support this assertion. For example, regular customers can become an entrepreneur's friends and provide encouragement and referrals (Nambiar et al., 2020). Similarly, peers can provide support and useful recommendations (Nambiar et al., 2020). Government involvement is vital for converting knowledge and skills into entrepreneurship (Yoon et al., 2018) and government support influences strategic overhauling (Shu et al., 2019). Similarly, non-government organizations play a central role in promoting innovation and entrepreneurship (Ritchie, 2016). Sonck et al. (2017) have recommended including stakeholders in research and development, to extract the usefulness of innovation products. In this setting, service intermediaries provide start-ups with relevant knowledge (Smeltzer et al., 1991).

Entrepreneurs obtain legitimacy through these external stakeholders, which allows them to obtain the resources they need (Vershinina et al., 2020). Networking with external stakeholders provides entrepreneurs with legitimacy amongst internal stakeholders, which further improves innovation and performance of the ventures (Vershinina et al., 2020). Based on the findings highlighted above, the following relationship has been hypothesized.

*H*_4_ *: There is a positive association between external stakeholder relationships and entrepreneurial success*

### Moderating Role of Digital Technology Adoption

Technology can be the base for creation of small businesses or development of existing enterprises (Linton and Solomon, 2017). The steady introduction of digital content into a large variety of products and services has made available a larger pool of opportunities for entrepreneurs (Davidson and Vaast, 2010; Porter and Heppelmann, 2014; Nambisan, 2017; Srinivasan and Venkatraman, 2018; von Briel et al., 2018b). Since information technology (IT) processes are conducted using a combination of technologies (Zhao et al., 2017) they enable an organization to record, process and exchange data effectively (Gupta and Misra, 2016). Organizations can use this data to extract information for multiple purposes, including faster and higher-quality decision making (Coreynen et al., 2017). Aligned with this, Ughetto et al. (2019) have observed that digitally mediated platforms provide quicker access to opportunities for entrepreneurship. Similarly, Yetis-Larsson et al. (2015) have found that digital technology can provide faster communication, free from time and space restrictions. This provides access to international markets, for businesses (Hansen, 2019). So, IT provides multiple advantages to an enterprise and plays a central role in entrepreneurs' success (Yunis et al., 2018). In the context of China, digitalization has helped to revitalize entrepreneurship (Hansen, 2019).

Digital technology adoption is defined as the business-related use of computer-based solutions (Bharadwaj, 2000; Urbinati et al., 2020). Through these technologies, businesses can generate value (Yoo et al., 2010; Kammerlander et al., 2018; Soluk and Kammerlander, 2021) which can help them become entrepreneurial (Nambisan, 2017; Autio et al., 2018). So, it can be stated that technology has a direct and positive impact on entrepreneurship success.

This technology adoption can provide several benefits including lower costs, higher revenue, competitive advantages and the opportunity to build new business models (Bharadwaj, 2000; Yoo et al., 2010; Remane et al., 2017; Soluk et al., 2021). Soto-Acosta et al. (2016) have stated that IT is considered irreplaceable for increasing the pace of innovation. Crittenden et al. (2019) have stated that encouraging information and communication technology (ICT) use to support entrepreneurs is essential. Entrepreneurs who use new technologies are more likely to achieve improved organizational performance (Stoneman and Kwon, 1996) because technology can also help new ventures in solving problems they face, such as lack of social and economic capital (Morse et al., 2007). In this setting, Soluk et al. (2021) have observed that positive effects of family and community networking become stronger when entrepreneurs adopt digital technologies. Similarly, IT processes have been found to positively impact innovation by supporting supply chain collaboration (Liao and Barnes, 2015). In harmony with this, Venkatesh et al. (2017) have discovered that interaction of ICT with social networks plays a central role in promoting entrepreneurial actions and success. Further, technology use may be particularly relevant for developing countries (Soluk et al., 2021). An entrepreneur can utilize digital technology for quickly testing the suggestions offered by family members (Hossain and Rahman, 2018), which shows that technology can strengthen the positive effects of support mechanisms on entrepreneurial success. So, technology can be expected to play a moderating role in the relationships between support and entrepreneurial success. Specifically, it can be expected to strengthen the positive associations between different types of support and entrepreneurial success.

However, breakthrough technologies may adversely affect new product introduction if the technology breakthrough nature is at a very high level (Ardito et al., 2020). So, businesses should strike a balance between breakthrough and incremental technologies (Ardito et al., 2020). Digital technology can also generate role conflict for an entrepreneur because the person has to act as both a venture leader and a digital platform follower (Nambisan and Baron, 2019). This conflict leads to stress which negatively affects organizational performance (Nambisan and Baron, 2019). Another problem associated with technology is that access to more information means more filtering and organizing are needed, to avoid fake or misleading information (Hansen, 2019). Businesses also need to take into account that IT infrastructure and digital technologies require large amounts of investment (Hansen, 2019). Further, there are many types of software and hardware so an entrepreneur needs to obtain the required skills and experience before technologies can be deployed (Hansen, 2019). Based on the research discussed above, the following hypotheses have been developed.

*H*_5_ *: There is a positive relationship between digital technology adoption and entrepreneurial success*

*H*_5_ *: The positive associations of (a) family support, (b) business partner support, (c) community support and (d) external stakeholder relationships with entrepreneurial success are moderated by digital technology adoption so that these associations are stronger at higher levels of technology adoption*

**Figure 1:**
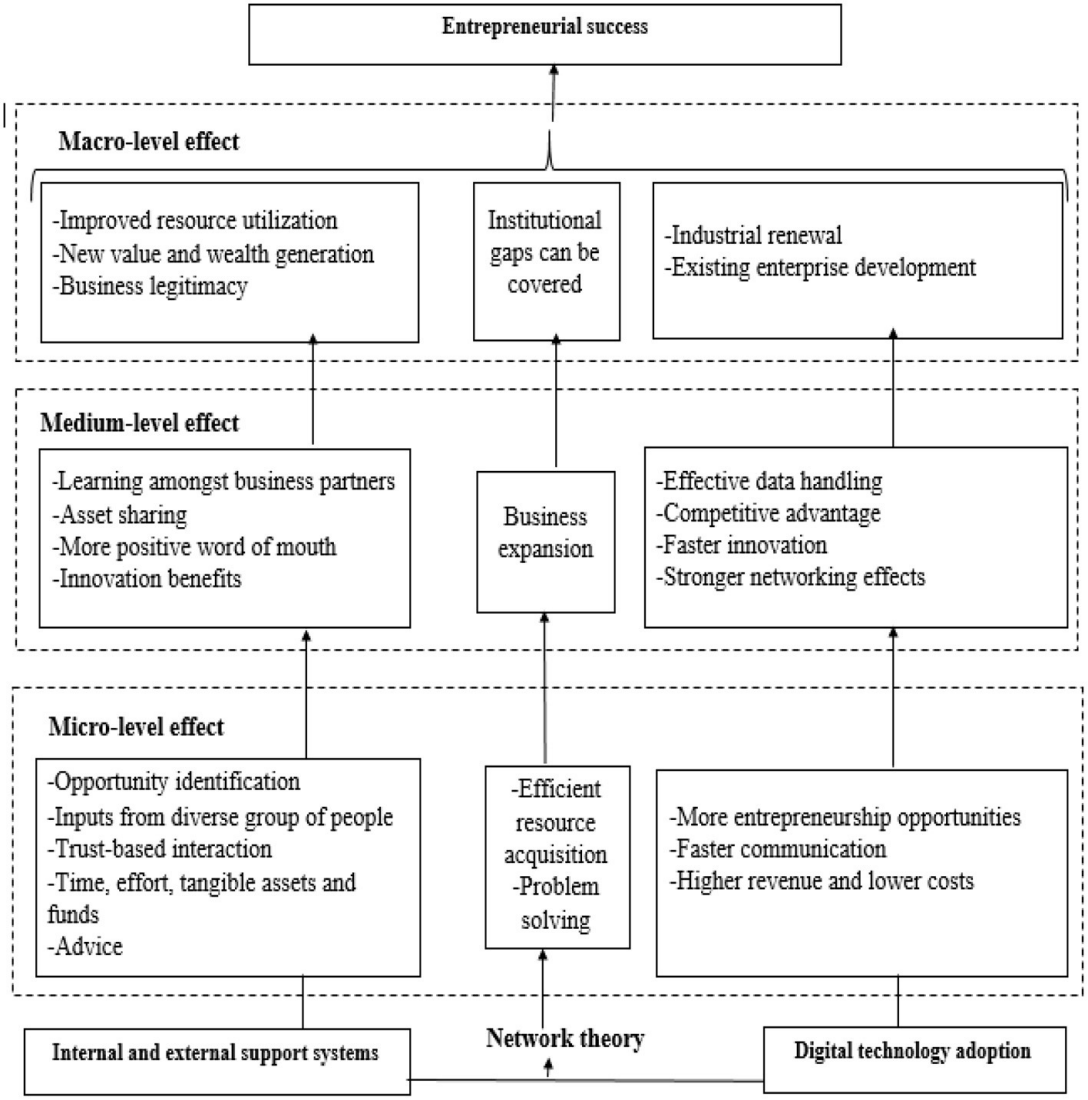
The association among internal and external support mechanisms, digital technology adoption and entrepreneurial success from network theory perspective.

Based on all of the above proposed hypothesis and the theoretical foundation the conceptual association among variables is presented below in Figure 1.

## Materials and Methods

Data were collected from the owners of entrepreneurial startups located in Jiangsu Province, China. The present study was directed using a cross-sectional method. The primary reason of choosing the owners of entrepreneurial start-ups for data collection was to deeper explore the small-scale business organizations that has experienced accelerating growth patterns in the contemporary time (Breznitz and Zhang, 2019). China is overtaking the world as the new Silicon Valley (Klingler-Vidra, 2019). Especially, in the last decade the East Asian region has experienced rapid and dynamic growth in the context of entrepreneurial start-ups (Hemmert et al., 2021). Indeed, the Chinese economy has scored the second highest position in terms of global venture capital (Dutta et al., 2020). In 2017, more than 100 Chinese start-ups and 34 unicorns were listed on global stock market whereas five years down the road there are more than 250 Chinese start-ups who have made their position to global stock markets (Lee and Shen, 2020).

There are seven key reasons that has made China a favorable location for entrepreneurial start-up settings. Firstly, China venture capital funding is ranked as one of the best in the world (Huang et al., 2020). The Chinese governmental authorities have guidance funds at the national, provisional and municipal level (Zhang et al., 2021). The Chinese government has taken a prominent role in business lawmaking, from tax incentives to financial laws, in order to make China the most attractive destination to start a business (Basu and Ray, 2021). Entrepreneurs benefit from lower tax rates, lower tariffs, easier business registration, easier importing and exporting processes, and minority investor protection (Alon et al., 2019). China rated 31st out of 190 nations in the World Bank's “Ease of Doing Business” report in 2020, and for the 2nd year in a row, it joined the top 10 most improved economies (FINANCE, 2020). Thirdly, Chinese policymakers have emphasized consumerism as the mantra for China's emergence as a global economic force during the last decade (Gu et al., 2017). China is now the world's fastest-growing consumer market, with the country's middle class fueling the growth (Atherton, 2021). In 2019, the country's consumer goods retail sales were 3.81 trillion yuan ($540 billion), reducing the $280 billion gap between China and the United States (Wang et al., 2020). Fourthly, in China's startup landscape, the proliferation of entrepreneurial spaces seems fundamental. In China, there are already 4,300 creative spaces, 3,300 incubators, and 400 accelerators, with government incentives expected to expand these numbers every year (Cooke, 2017). Fifthly, China is among the few nations in the world with several internationally competitive startup ecosystems (Barton et al., 2017). The most noteworthy Chinese megacities that have cultivated the best startup environments in the world include Beijing, Shanghai, Shenzhen, Hangzhou, and Wuhan (Ye and Björner, 2018). Sixth, running a startup in China is capital, time, and resource efficient with enough investment, active use of technology in business, and fair business ethics (Hou et al., 2020). Lastly, among emerging countries, China enjoys a labor cost advantage (Cui and Lu, 2018).

### Conceptual Model

**Figure 2:**
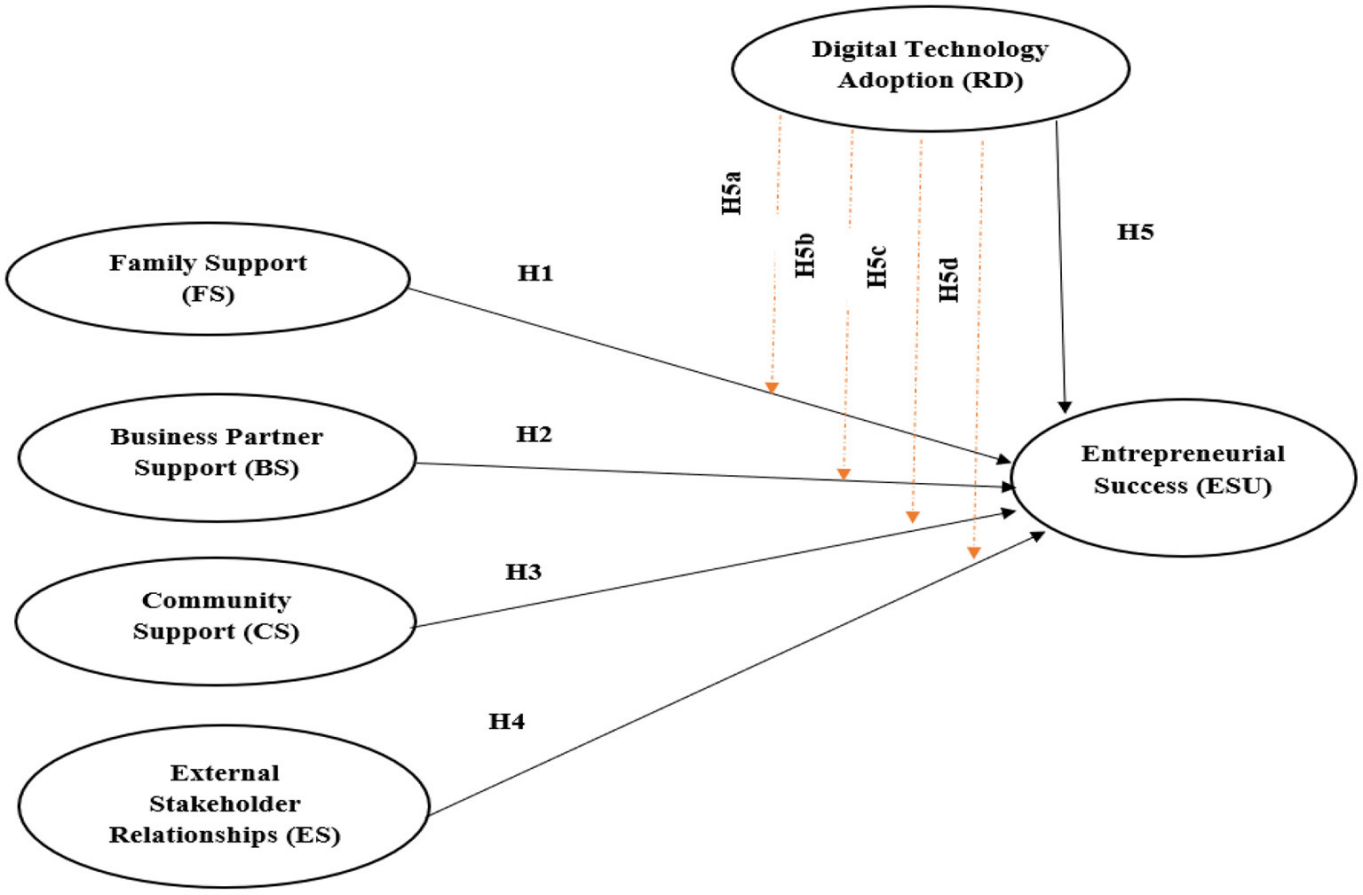
Conceptual model. BS, Business partner support; FS, Family support; CS, Community support; ES, External stakeholder relationship; ESU, Entrepreneurial success; RD, Digital Technology adoption.

In this study, Chinese participants were taken as the research subject to investigate the relationship among the variables, including business partner support (BS), family support (FS), community support (CS), external stakeholder relationship (ES), entrepreneurial success (ESU) and moderating role of digital technology adoption (RD). According to the hypotheses proposed above, we created the conceptual model shown in Figure 2.

### Pilot Survey and Instrumental Design

Based on validated scales, we built a preliminary questionnaire. We altered and merged these measures before conducting the final survey. Twenty randomly selected volunteers, with entrepreneurial start-ups in Jiangsu, completed the pilot questionnaire and checked its layout as well as content validity. We constructed the final questionnaire to improve the instrument's relevance and readability. The cross-sectional questionnaire included 32 items, based on the pilot survey and observations of Chinese language expression patterns.

### Sampling Technique and Demographic Information

Taking into consideration the focus of this study, we included only entrepreneurs in our sample. Individuals who owned entrepreneurial start-ups in Jiangsu were selected, using a non-probability (convenience) sampling method. The sample size is 350, recommended by Mason (2010) using the formula Z^2*^p (1–p)/e^2^, where z = 1.6384, *p* = 0.25 and e^2^ = 0.0016. We contacted the entrepreneurs and presented our research purpose. After obtaining permission to conduct the study, we provided the questionnaire and requested them to fill and email it. A Computer-Assisted Web Interview method was used for data collection, in which respondents use computers to complete questionnaires without being directed by interviewers (Sowa et al., 2015). We completed this data collection between January and June 2021. If the target population has more than 4,000 members, a minimum sample size of 500 is sufficient (Krejcie and Morgan, 1970). To obtain more responses, we distributed 500 online questionnaires for entrepreneurs which could be completed in spare time. Based on the findings of Hair et al. (2017a,b) and Cohen's power theory, we have assessed sample size adequacy. To confirm the sample's statistical strength, we used the G^*^power *post-hoc* test for exogenous factors (with a significance level of 0.05), an effect size of 0.15, and a sample size of 380. Results of the G^*^power *post-hoc* analysis revealed that statistical strength was substantially higher than the required value of 0.8 (Faul et al., 2009).

Since Chinese is the official language in China, the first set of questionnaires was written in Chinese. The entrepreneurs' participation was voluntary and the confidentiality of their responses was guaranteed (Podsakoff et al., 2003), to lower the risk of common method bias (CMB). There were 500 questionnaires and 80 were rejected, due to missing or incorrect responses. Three hundred eighty questionnaires were retained for data analysis, so the response rate was 76%.

We calculated the following statistics based on demographic data. Males completed 197 (51.8%) of the questions, while females completed 183 (48.2%). The age range was 18–55 years, with a mean of 2.53 and standard deviation of 1.47. The age group of 25–30 years had the highest response rate (45%). It was followed by the group of 30–45 years (25%). 20% of the respondents were over 45 years and 10% were under 25 years. The most common education level was a bachelor's degree (62.18%). In terms of regional distribution, 162 (42.63%) respondents were from Nanjing, 103 (27.11%) were from Suzhou, 53 (13.95%) were from Wuxi, 38 (10%) were from Zhenjiang and the remaining were from Changzhou.

### Measures

All items (except the number of employees) were presented on a 5-point scale, from 1 (strongly disagree) to 5 (strongly agree). For the number of employees, there were 5 options (from 0–5 to 21–25). Family support was measured using 4 items (Powell et al., 2005). A sample item was *My family gives me useful feedback about my ideas concerning my business*. For this construct, Cronbach's alpha was 0.933. Community support was captured using 6 items (Niehm et al., 2008). One of the items was *The people of this community truly care about the fate of this business*. For this construct, Cronbach's alpha was 0.952. Business partner support was measured through 9 items (Stanko and Henard, 2017; Bogers et al., 2018). A sample item was *the extent of use of suppliers as a source of external knowledge*. Cronbach's alpha was 0.943. External stakeholder relationships were captured through 8 items (Mazur and Pisarski, 2015). One of the items was *I am satisfied with the benefits I receive from my stakeholder relationships*. Cronbach's alpha was 0.936. Digital technology adoption was measured using 4 items (Srinivasan and Venkatraman, 2018). A sample item was *We have implemented digital tools in all our business processes*. For this construct, Cronbach's alpha was 0.904. Entrepreneurial success was captured through 4 items (Cooper and Dunkelberg, 1986; Chaganti et al., 1996; Bosma et al., 2000; Caliendo and Kritikos, 2008; Fried and Tauer, 2009). A sample item was the *number of employees*. Cronbach's alpha was 0.933.

## Results

### Data Analysis Technique

To test the hypotheses, this study has used SEM. When the sample size is 200 or above this, SEM should be applied (Kline, 2005). SEM is a statistical method that comprises mathematical and statistical approaches for examining data, to identify relationships between variables (Purwanto et al., 2021). Employing the software SmartPLS v3, this study has conducted PLS-SEM (Hair et al., 2014; Sarstedt et al., 2019). This software is useful for measuring mediating and moderating effects in the same path model, and is suitable for the exploratory nature of study analysis (Dash and Paul, 2021). In recent years the number of published articles using PLS-SEM increased significantly, compared with CB-SEM (Hair et al., 2017b). PLS-SEM is now applied in many social science areas, including organizational management (Sosik et al., 2009). SmartPLS is a user-friendly software package which requires little technical knowledge about the method (Ringle et al., 2005, 2015). This software's strength comes from its ability to check prediction applications, build theories and provide explanations (Chin, 1998). When using PLS-SEM researchers benefit from the high statistical power, compared with CB-SEM (Reinartz et al., 2009; Hair et al., 2017b). This means that PLS-SEM is more likely to highlight relationships as significant when they are indeed present in the population (Sarstedt and Mooi, 2014).

### Measurement of the Model

It is essential to check the reliability and validity of measurement tools utilized. Construct reliability and composite reliability have been checked (Brown, 2002). The values are provided in Table 1. All are higher than the suggested threshold value of 0.70 (Nunally and Bernstein, 1978). The average variance extracted (AVE) values, given in Table 1, have been used to assess convergent validity. The values are acceptable for the full model, compared to the generally used threshold value of 0.5 (Henseler et al., 2016). Multicollinearity has been checked (Aiken et al., 1991). An outer Variance Inflation Factor (VIF) is acceptable if it does not exceed 5 (Ringle et al., 2015) or even 10 (Hair et al., 1995). VIF values for all constructs are <5 and, therefore, acceptable (Table 1).

**Table 1:** Factor loadings.

**Constructs**	**Loadings**	**Cronbach's Alpha (CA)**	**Composite Reliability (CR)**	**Average Variance Extracted (AVE)**	**Variance Inflation Factor (VIF)**
Business Partner Support (BS)		0.943	0.955	0.781	
BS 1	0.843				2.801
BS 2	0.790				2.249
BS 3	0.877				3.290
BS 4	0.936				5.653
BS 5	0.914				4.575
BS 6	0.934				5.674
Family Support (FS)		0.933	0.952	0.832	
FS 1					3.062
FS 2					3.419
FS 3					3.877
FS 4					3.618
Community Support (CS)		0.952	0.963	0.838	
CS 1	0.945				0.945
CS 2	0.929				0.929
CS 3	0.916				0.916
CS 4	0.870				0.870
CS 5	0.915				0.915
External Stakeholder Relationships (ES)		0.936	0.946	0.661	
ES 1	0.760				0.760
ES 2	0.817				0.817
ES 3	0.846				0.846
ES 4	0.821				0.821
ES 5	0.756				0.756
ES 6	0.815				0.815
ES 7	0.871				0.871
ES 8	0.847				0.847
ES 9	0.776				0.776
Digital Technology Adoption (RD)		0.904	0.933	0.777	
RD 1	0.904				0.904
RD 2	0.895				0.895
RD 3	0.849				0.849
RD 4	0.876				0.876
Entrepreneurial Success (ESU)		0.933	0.952	0.833	
ESU 1	0.905				0.905
ESU 2	0.891				0.895
ESU 3	0.922				0.849
ESU 4	0.931				0.876

### Reliability and Validity Test

Discriminant validity has been assessed using the Fornell Larcker criterion as well as the Heterotrait Monotrait ratio (HTMT). Both criteria are commonly recognized and other researchers have employed them (Henseler et al., 2016; Neneh, 2019a,b). Discriminant validity is defined as the square root of AVE (Fornell and Larcker, 1981). HTMT values must be <0.85 (Henseler et al., 2016). The highest HTMT value is 0.546 which shows that all constructs possess discriminant validity. Tables 2, 3 present the discriminant validity values.

**Table 2:** Discriminant validity (Fornell Larcker).

	**BS**	**CS**	**ES**	**ESU**	**FS**	**RD**
BS	0.884	-	-	-	-	-
CS	0.396	0.915	-	-	-	-
ES	0.389	0.410	0.813	-	-	-
ESU	0.412	0.483	0.456	0.912	-	-
FS	0.380	0.517	0.401	0.394	0.912	-
RD	0.353	0.252	0.390	0.386	0.251	0.881

*Values in diagonals are square roots of AVE. Values under diagonals are correlations. BS, Business Partner Support; FS, Family Support; CS, Community Support; ES, External Stakeholder Relationships; RD, Digital Technology Adoption; ESU, Entrepreneurial Success*.

**Table 3:** Discriminant validity (HTMT).

	**BS**	**CS**	**ES**	**ESU**	**FS**	**RD**
BS	**-**	**-**	**-**	**-**	**-**	**-**
CS	0.416	-	-	-	-	**-**
ES	0.405	0.433	-	-	-	**-**
ESU	0.437	0.512	0.485	-	-	**-**
FS	0.405	0.546	0.425	0.420	-	**-**
RD	0.380	0.273	0.425	0.417	0.273	**-**

*BS, Business Partner Support; FS, Family Support; CS, Community Support; ES, External Stakeholder Relationships; RD, Digital Technology Adoption; ESU, Entrepreneurial Success*.

### Common Method Bias

Harman's one-factor test has been conducted to detect Common Method Bias (CMB) (Harman, 1976). This bias exists if any single factor explains more than half of the total variance (Podsakoff et al., 2003). All of the factors have been combined to form one factor, which explains 25.82% of the variance. This means that CMB does not exist.

### Structural Model

**Figure 3:**
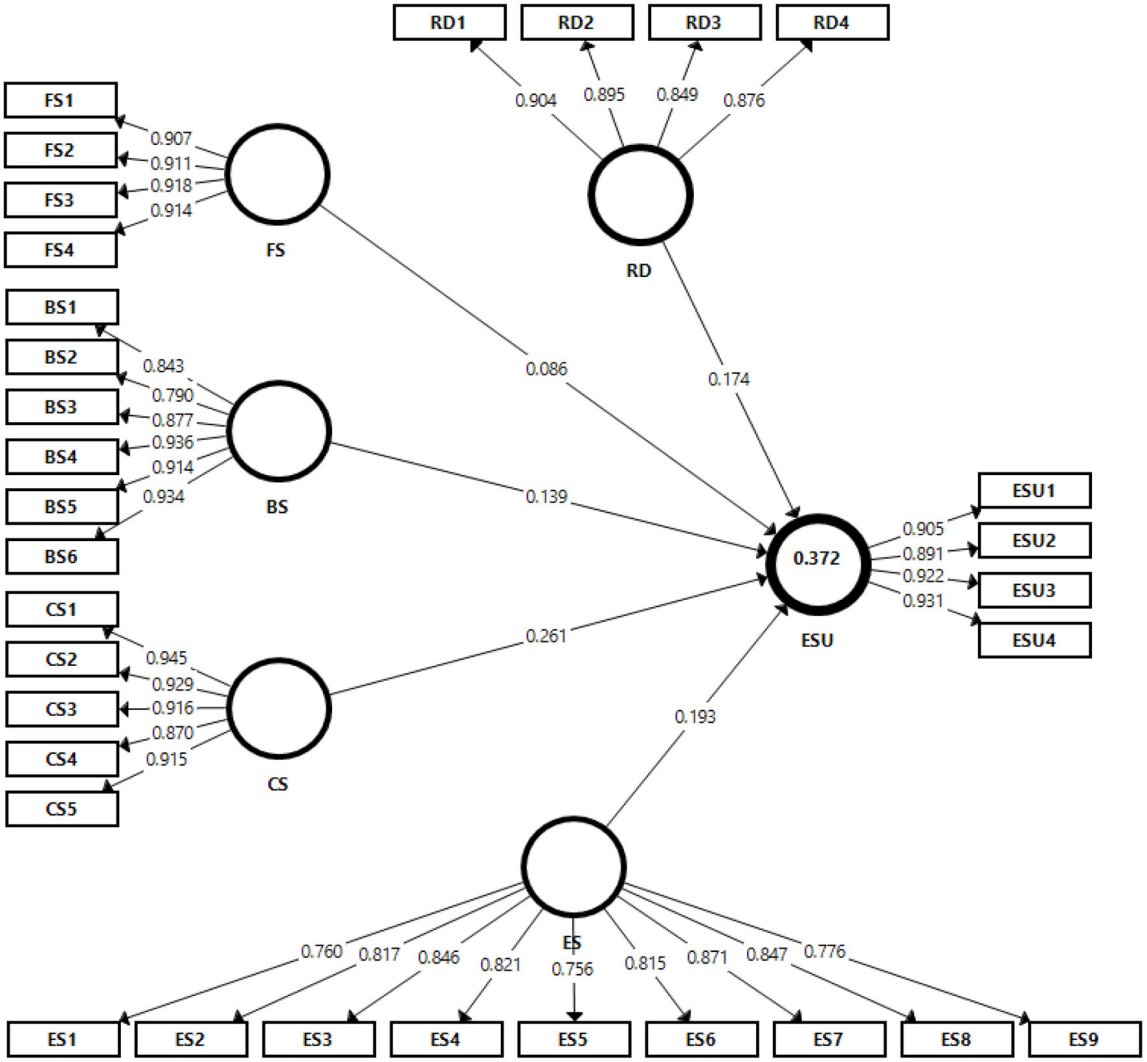
Structural model. BS, Business partner support; FS, Family support; CS, Community support; ES, External stakeholder relationship; ESU, Entrepreneurial success; RD, Digital Technology adoption.

SmartPLS v3 and the PLS algorithm approach have been deployed to analyse the structural model (Cheah et al., 2020). The standardized root mean square residual value has been used to assess model fit, with a suggested value of 0.08 (Henseler et al., 2016). This model's value is 0.044, indicating the model's overall fitness. Figure 3 presents the *R*^2^ value, which means that this model explains 37.2% of the variance in entrepreneurial success. In past research, a model based on entrepreneurial success has explained only 20–40% of the variance in this success (Staniewski and Awruk, 2019).

### Hypothesis Testing

All hypotheses have been tested using bootstrapping. All the direct-effect hypotheses have been accepted. These results are shown in Table 4. Family support has a significant and positive impact on entrepreneurial success (β = 0.097^*^, *t* = 2.159, *p* < 0.05), providing support for H1. Business partner support also has a significant and positive influence on entrepreneurial success (β = 0.201^**^, *t* = 4.031, *p* < 0.01) so H2 is supported. There is a positive and significant influence of community support on entrepreneurial success (β = 0.250^**^, *t* = 5.137, *p* < 0.01) so H3 has been accepted. There is a positive and significant influence of external stakeholder relationships on entrepreneurial success (β = 0.149^**^, *t* = 2.774, *p* < 0.01) so H4 has also been accepted. A positive and significant direct effect of digital technology adoption on entrepreneurial success has been found (β = 0.208^**^, *t* = 3.971, *p* < 0.01), supporting H5.

**Table 4:** Structural model estimates.

**Hypotheses**	**Relationships**	**Standardized paths (β)**	**T-statistics**	***P*** **-values**	**Hypotheses accepted/not accepted**
H1	FS -> ESU	0.097^*^	2.159	0.031	Accepted
H2	BS -> ESU	0.201^**^	4.031	0.000	Accepted
H3	CS -> ESU	0.250^**^	5.137	0.000	Accepted
H4	ES -> ESU	0.149^**^	2.774	0.006	Accepted
H5	RD -> ESU	0.208^**^	3.971	0.000	Accepted

** *p < 0.01*;

* *p < 0.05. BS, Business Partner Support; FS, Family Support; CS, Community Support; ES, External Stakeholder Relationships; RD, Digital Technology Adoption; ESU, Entrepreneurial Success*.

### Moderating Effect

**Figure 4:**
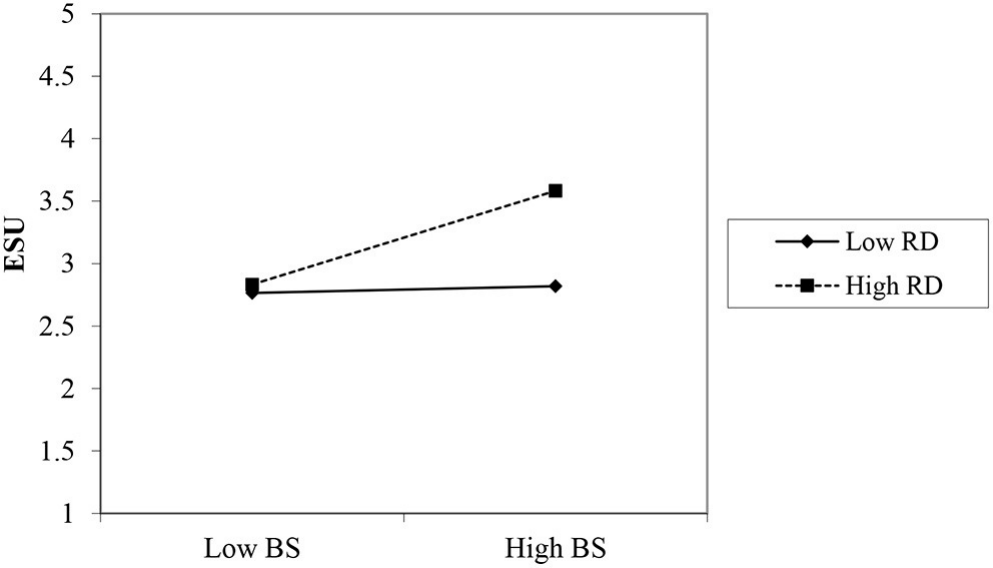
Interaction graph RD*BS and ESU.

The Table 5 shows the moderating effects. H5a is about the moderating role of digital technology adoption in the relationship between family support and entrepreneurial success. Technology adoption strengthens the positive relationship but this effect is insignificant (β = 0.042, *t* = 0.882, p>0.05). H5b is about the moderating role of digital technology adoption in the relationship between business partner support and entrepreneurial success. Technology adoption strengthens the positive relationship and this effect is significant (β = 0.174^*^, *t* = 3.343, *p* < 0.01). The interaction is shown in Figure 4. H5c is about the moderating effect of digital technology adoption on the relationship between community support and entrepreneurial success. Technology adoption has a significant effect but it weakens the positive relationship (β =−0.118^*^, *t* = 3.343, *p* < 0.01). H5d is about the moderating effect of digital technology adoption on the relationship between external stakeholder relationships and entrepreneurial success. Technology adoption weakens the positive relationship but this effect is insignificant (β =−0.042, *t* = 0.442, *p* > 0.05). Therefore, H5b has been accepted.

**Table 5:** Moderating effects.

**Hypotheses**	**Relationships**	**Standardized paths (β)**	**T-statistics**	***P*** **-values**	**Hypotheses accepted/not accepted**
H5a	RD^*^FS and ESU -> ESU	0.042	0.882	0.378	Not accepted
H5b	RD^*^BS and ESU -> ESU	0.174^**^	3.343	0.001	Accepted
H5c	RD^*^CS and ESU -> ESU	−0.118^*^	2.186	0.029	Not accepted
H5d	RD^*^ES and ESU -> ESU	−0.024	0.442	0.658	Not accepted

** *p < 0.01*;

* *p < 0.05. BS, Business Partner Support; FS, Family Support; CS, Community Support; ES, External Stakeholder Relationships; RD, Digital Technology Adoption; ESU, Entrepreneurial Success*.

## Discussion

This study has examined the effects of family support, business partner support, community support and external stakeholder relationships on entrepreneurial success. It has also analyzed the role of digital technology adoption in these relationships. Hypothesis 1 is accepted, showing that family support has a positive influence on entrepreneurial success. Soluk et al. (2021) also found a positive relationship between family support and entrepreneurship. They highlighted the fact that families provide valuable support to entrepreneurs in the form of time, effort, tangible assets and funding. So an entrepreneur's family is viewed as an important stakeholder of the enterprise (Aldrich and Cliff, 2003; Bruque and Moyano, 2007; Duran et al., 2016; Hatak et al., 2016). Hypothesis 2 is accepted, showing that business partner support positively affects entrepreneurial success. In harmony with this, Thorgren et al. (2012) have observed that as partner fit improves in business networks there is an improvement in entrepreneurship as well. Research has shown that businesses benefit from building and maintaining such relationships (Powell et al., 2005; Kumar and Pansari, 2016). Hypothesis 3 is accepted, which means that community support and entrepreneurial success have a positive association. This result is aligned with a study by Kwon et al. (2013) in which it has been found that community support enhances entrepreneurship. Entrepreneurs utilize such networks to acquire resources and achieve success (Chen et al., 2015). Hypothesis 4 is accepted, showing that external stakeholder relationships and entrepreneurial success have a positive association. External stakeholder support, as far as we know, has not been explored as a part of a detailed model in past research. So, our study has made an important theoretical contribution by including this factor. H5b is also accepted, showing that digital technology adoption strengthens the positive relationship between business partner support and entrepreneurial success. This finding is supported by earlier research that has found that interaction of ICT with social networks plays a central role in promoting entrepreneurial actions and success (Venkatesh et al., 2017). It is also supported by research which has found that the positive effects of networking become stronger when entrepreneurs adopt digital technologies (Soluk et al., 2021). All the results of this study provide support for network theory. H5c is not accepted because digital technology adoption has a negative effect. Based on existing research, a positive effect was predicted.

### Theoretical Implications

First, this study has added to earlier research by analyzing the role of external stakeholder relationships in entrepreneurial success (Soluk et al., 2021). Second, the authors have added to the inadequate research on how family support influences entrepreneurship success (Staniewski and Awruk, 2021). Third, the findings are different from those obtained by Soluk et al. (2021) because a negative relationship between business partner support and entrepreneurship has not been found. Fourth, we have built a detailed framework that includes family support, business partner support, community support, external stakeholder relationships, digital technology adoption and entrepreneurial success. As far as we know, such a model has not been explored in past studies. Fifth, the authors have responded to calls for research by analyzing the effects of technology on relationships between support systems and entrepreneurial success (Nambisan et al., 2017; Majchrzak et al., 2018). Sixth, this research has made a contextual contribution by investigating entrepreneurship in a developing country (Bruton et al., 2008; Chatterjee et al., 2018). Seventh, the study has added to the insufficient knowledge about digital technology's role in developing contexts (Soluk et al., 2021). Eighth, the findings have provided support for network theory (Watson, 2007).

### Practical Implications

This study has provided valuable practical implications as well. It has revealed the sources of support for boosting entrepreneurial success. Individuals who are interested in setting up their own businesses can use these findings, to acquire support from the appropriate sources. The study has also highlighted digital technology's positive role in entrepreneurial success. So, the findings will encourage entrepreneurs to utilize technologies to achieve success. The findings are useful for policy makers as well, for designing strategies to promote entrepreneurship in developing countries. For example, authorities should incorporate entrepreneurial education in academic programs. This research also shows that ties should be strengthened between the government, industries and the education sector. This is because government support and education have been found to be valuable for promoting entrepreneurship.

## Limitations and Future Research Directions

Our research has some limitations which can be viewed as opportunities for future research. First, this is a cross-sectional study so we do not claim to have discovered causal relationships. Longitudinal studies are recommended for future studies because these are more appropriate for detecting causality. Second, our data has been taken from only the Chinese entrepreneurship sector which means there is limited applicability of our findings. Different settings, such as large-scale businesses or Western cultures, should be explored to find out whether these associations hold for those. Third, the model can be expanded by adding mediating variables such as big data—driven processes as well as moderating variables such as digitized mass production (Hyers, 2020; Nemţeanu and Dabija, 2021; Riley et al., 2021; Wade and Vochozka, 2021). This approach will generate useful and interesting insights. Smart manufacturing systems are an important area for entrepreneurs so future studies should investigate the effects of support mechanisms on entrepreneurship in the context of smart manufacturing and smart factories (Kovacova and Lăzăroiu, 2021; Suler et al., 2021). The role of artificial intelligence in decision-making and performance of international businesses should also be analyzed in future research (Vătămănescu et al., 2020; Cunningham, 2021). Fourth, digital technology adoption was found to weaken the positive relationship between community support and entrepreneurial success. This is a surprising finding and should be investigated in future studies. Fifth, there could be mediated moderation between support sources and entrepreneurial success and we suggest future researchers investigate this.

## Conclusion

The purpose of this research is to analyze associations of internal and external support mechanisms with entrepreneurial success, in the context of China. The role of digital technology, as a moderator, has also been investigated. The network theory has been utilized to support this framework. Data has been obtained from 500 entrepreneurs in Jiangsu, a province in China. All hypotheses have been tested using PLS-SEM. It has been found that family support, business partner support, community support and external stakeholder relationships have positive effects on entrepreneurial success. It has also been discovered that digital technology adoption strengthens the positive relationship between business partner support and entrepreneurial success.

This research has made a theoretical contribution by including both internal and external factors in the model. The study has made an empirical contribution also, by applying PLS-SEM in SmartPLS v3 for data analysis. It has generated multiple theoretical and practical implications. No comprehensive study has investigated the effects of external stakeholder relationships on entrepreneurial success and the moderating impact of digital technology adoption, using the network theory. The findings will help entrepreneurs and policy makers, as the study has highlighted relevant support mechanisms as well as the role of digital technology. These support mechanisms help build an environment that is conducive to entrepreneurship. Adoption of digital technology is critical for ensuring entrepreneurial success in today's dynamic business world. In the context of China, after the Belt and Road Initiative, there has been growth of university-enterprise alliances for promotion of local as well as international talent. Specifically, in Jiangsu, the Jiangsu University Belt and Road Industry-Education Integration Institute has played a significant role in generating and supporting both internal and external support mechanisms to promote entrepreneurial success. This study has also created opportunities for future researchers.

## Data Availability Statement

The raw data supporting the conclusions of this article will be made available by the authors, without undue reservation.

## Ethics Statement

Ethical review and approval was not required for the study on human participants in accordance with the local legislation and institutional requirements. The patients/participants provided their written informed consent to participate in this study.

## Author Contributions

CY and SF contributed to developing the theoretical framework, data analysis, and overall writing of the manuscript. AA, VF, and MA contributed to data collection and the writing, editing, and organization of the manuscript. All authors contributed to the article and approved the submitted version.

## Funding

This article was supported by the following research funds: General Program of The Chinese Society of Academic Degrees and Graduate Education Research Fund, program title, “Research and Practice of the Promotion the Quality of Overseas Engineering Postgraduate Students Education Through the Integration between Industry and University” (No. 2020MSA350). China Education Association for International Exchange Research Fund, program title, “Research and Practice of the Promotion the Quality of Overseas Engineering Students Education Through the Integration among government, Industry and University” (No. 际协研2021-008). Major Program of The Chinese Society of Academic Degrees and Graduate Education Research Fund, program title, “Exploration and Practice of the Cultivation Mode of the Students from Belt and Road Countries” (No. 2020ZA1013). The Key Project of Philosophy and Social Science Research in Colleges and Universities in Jiangsu Province, program title, “Research on the Academic Achievement of the Undergraduate Engineering Students from Belt and Road Countries” (No. 2018 SZJDI172).

## Conflict of Interest

The authors declare that the research was conducted in the absence of any commercial or financial relationships that could be construed as a potential conflict of interest.

## Publisher's Note

All claims expressed in this article are solely those of the authors and do not necessarily represent those of their affiliated organizations, or those of the publisher, the editors and the reviewers. Any product that may be evaluated in this article, or claim that may be made by its manufacturer, is not guaranteed or endorsed by the publisher.
