# Supplementary material for: The Influence of Internal and External Stakeholder Mechanisms on Entrepreneurial Success: The Moderating Role of Digital Technology Adoption
Source: Front Psychol. 2022 Feb 3;12:821725. doi: 10.3389/fpsyg.2021.821725 (PMC8850971; doi:10.3389/fpsyg.2021.821725)
Supplement: Supplementary file 1 [file Data_Sheet_1.pdf]

Appendix A

All the items are measured using five-point Likert scales ranging from 1 strongly disagree to 5 strongly agree

Variable Name	Items	1	2	3	4	5
**FS1**	My family gives me useful feedback about my ideas concerning my business					
**FS2**	When I'm frustrated by my business, someone in my family tries to understand					
**FS3**	Family members often go above and beyond what is normally expected in order to help my business succeed					
**FS4**	Family members often contribute to my business without expecting to be paid					
**CS1**	The people of this community truly care about the fate of this business					
**CS2**	If given a chance, you would brag about this community as a good place to locate a business					
**CS3**	How satisfied are you with the amount of support your business gets from your community?					
**CS4**	As a business owner or manager, are you willing to expend resources to help this town?					
**CS5**	If you feel like talking, you usually can find someone in the community to talk with					
**BS1**	My firm get support from consultants, commercial laboratories or private R&D?					

**BS2**	My firm get support from universities or other higher education institutions.
**BS3**	My firm get support from public research institutions.
**BS4**	My firm get support from conferences, trade fairs or exhibitions.
**BS5**	My firm get support from industry associations.
**BS6**	My firm get support from competitors, suppliers and customers.
**ES1**	I am satisfied with the benefits I receive from my stakeholder relationships
**ES2**	My feelings toward my stakeholders are positive
**ES3**	I feel enthusiastic about my stakeholder relationships
**ES4**	All in all, I am satisfied with my stakeholder relationships
**ES5**	My stakeholder relationships always achieve their objectives
**ES6**	My stakeholder relationships provide me with everything that I need from the relationship
**ES7**	I think all my stakeholder relationships are successful
**ES8**	My stakeholder relationships achieve their purpose
**ES9**	I feel that my personal attributes (or characteristics) are well suited to establishing relationships with stakeholders.

**RD1**	I/We have implemented digital tools in all our business processes
**RD2**	Digital tools have had a very limited impact on our business operations
**RD3**	If there is great potential for digital tools in the business, we implement digital tools a lot
**RD4**	Digital tools have substantially changed our business processes
**ESU1**	Having greater number of employees leads to more business success
**ESU2**	Having owners’ involvement in business (number of hours) leads to more business success
**ESU3**	Profitability of the business leads to more business success
**ESU4**	Perceived chances of success influence business success
